# The serial mediation effects of parent-child relationships on juvenile delinquency: a nationwide analysis based on data from China's Recidivism Survey

**DOI:** 10.3389/fpsyg.2026.1811021

**Published:** 2026-04-23

**Authors:** Qianqian Chen, Weihong Sun

**Affiliations:** 1 Research Institute of Social Development, Southwestern University of Finance and Economics, Chengdu, China; 2 Faculty of Accounting, Tianfu College of Southwestern University of Finance and Economics, Chengdu, China

**Keywords:** deviant peer association, juvenile delinquency, parent–child relationships, self-control competence, serial mediation model

## Abstract

**Introduction:**

Juvenile delinquency has become an increasing concern in social governance, with the family, particularly the parent-child relationship, playing a central role in adolescent development and preventing antisocial behavior. However, the mechanisms linking parent-child relationships to delinquency, particularly in non-Western contexts, remain insufficiently understood.

**Methods:**

Using offender data from China's 2019 Recidivism Survey, this study examined whether parent–child relationships were associated with juvenile delinquency, defined as offending between ages 12 and 25. A serial mediation model was employed to test whether this association operated through self-control competence and deviant peer association, and whether the effect of self-control competence on juvenile delinquency differed by gender.

**Results:**

Results show that parent-child relationships are associated with juvenile delinquency directly and indirectly through self-control competence and deviant peer association. Relative to poor relationships, good and average parent-child relationships are associated with a lower likelihood of juvenile delinquency. Among mediators, deviant peer associations contribute the largest indirect effect. Gender moderation analyses indicate that self-control competence offers stronger protection against delinquency for males.

**Discussion:**

These findings contribute to integrated understanding of how family dynamics, individual self-regulation, and peer contexts are linked to juvenile delinquency and highlight the importance of supportive parent-child relationships for prevention.

## 1 Introduction

Juvenile delinquency represents a substantial threat to public safety and social stability, with an increasing trend toward earlier criminal involvement observed across various countries. Cross-national data underscore the scale and urgency of this issue. In the United Kingdom, knife-related offenses rose to 49,000 in 2022, with approximately 20% involving individuals aged 10–17, reflecting a significant increase from preceding years. Similarly, reports from the Tokyo Metropolitan Police indicate a 50.9% rise in juvenile delinquency cases in 2023 compared to the previous year, alongside a 54.1% increase in criminal law violations committed by youth. Comparable trends are observed in China. A White Paper released by the Supreme People's Procuratorate documents that the number of juvenile suspects aged 14–16 formally charged with crimes grew from 5,890 in 2016 to 9,317 in 2024, representing an average annual increase of approximately 5.82%. These patterns across diverse socio-legal contexts raise a central question: which factors and developmental processes drive the emergence of juvenile delinquency? A precise understanding of these mechanisms is essential for designing targeted and effective prevention and intervention policies.

The rise in juvenile delinquency and the shift toward earlier offending may reflect disruptions in normative socialization processes. A key source of such disruption is the family, particularly parent–child relationships. Research in neuropsychology and attachment theory suggests that emotional neglect and poor communication can impair prefrontal cortical development and increase emotional reactivity. These neuropsychological and relational impairments undermine self-regulation and empathic functioning, thereby elevating the risk of aggressive and antisocial behavior before children develop full decision-making capacity (Ramos-Galarza and Obregón, 2025). Consistent with this perspective, empirical studies indicate that delinquent youth are significantly more likely to come from neglectful family environments characterized by inadequate supervision, with the quality of the parent–child relationship consistently showing a negative association with deviant conduct (Worthen, 2011; Jacobsen and Zaatut, 2022). Consequently, within the landscape of juvenile delinquency, the family, particularly the parent–child relationship, constitutes a critical contextual factor: it is both closely associated with behavioral maladjustment and a central target for effective prevention and intervention strategies.

While existing research has provided valuable insights into juvenile delinquency, two key limitations remain. First, many studies rely on samples drawn from specific institutional or regional contexts (Simons and Sutton, 2021; Walters et al., 2023), which may constrain the generalizability of their findings to broader populations. Second, the literature has predominantly focused on the direct effects of familial, scholastic, psychological, and socioeconomic factors on delinquency (Baek et al., 2023; Jacobsen and Zaatut, 2022), while giving comparatively less attention to the indirect pathways through which parent–child relationships operate, particularly those mediated by individual traits, such as self-control, and external social networks, such as deviant peer associations. Although preliminary models such as “parental attachment → self-control → deviant behavior” have been proposed (Cho et al., 2017), the generalizability and robustness of such mediated pathways, especially among justice-involved youth and within culturally distinct settings like China, remain in need of further empirical examination.

To address these research gaps, this study utilizes large-scale micro-survey data from the 2019 Recidivism Survey administered by China's Ministry of Justice. We examine whether parent–child relationships are associated with juvenile delinquency, operationalized here as the first recorded offense occurring between ages 12 and 25. Grounded in prior theoretical and empirical work, we test a serial mediation model to investigate the pathways through which family dynamics, specifically parent–child relationships, relate to delinquency via two sequential mediators: self-control competence and deviant peer associations. Additionally, we assess whether the relationship between self-control and juvenile delinquency is moderated by gender. By analyzing nationally representative microdata from China's incarcerated youth population, this study offers novel empirical evidence on this hypothesized serial mediation mechanism within a non-Western, justice-involved context.

## 2 Literature review and hypotheses

### 2.1 Parent–child relationships and juvenile delinquency

The family, as the primary context of socialization, plays a central role in shaping individuals' cognitive, emotional, and behavioral development from early childhood onward, with parents constituting the core relational influence (Danisworo and Wangid, 2022). During adolescence, the parent–child relationship emerges as the most salient component of a youth's social environment, integrating emotional, structural, and authoritative dimensions that are continually negotiated through daily interaction (Keijsers et al., 2011; Koolaee et al., 2014; Merdović et al., 2024).

Accordingly, deficiencies in family socialization are widely recognized as a pivotal factor contributing to juvenile delinquency (Koolaee et al., 2014; Moitra and Mukherjee, 2012). Within the family context, the quality of the parent–child relationship serves as a strong predictor of delinquency risk (Hirschi, 2002; Worthen, 2011; Jacobsen and Zaatut, 2022). Empirical studies show that positive parent–child relationships not only mitigate adolescent psychological distress, such as depression and loneliness, but also significantly reduce involvement in deviant and criminal behaviors (Vieira et al., 2016; Smetana and Rote, 2019). These protective effects are primarily mediated through three interrelated mechanisms: (1) emotional bonding, which facilitates the internalization of prosocial norms and values (Liu et al., 2024); (2) behavioral supervision and monitoring, which constrains opportunities and situational motivations for delinquency (Demuth and Brown, 2004; Worthen, 2011); and (3) open communication and constructive engagement, which promote adaptive socioemotional development and reinforce conventional conduct (Tapia et al., 2018; Amran and Basri, 2020).

Conversely, parent–child conflict, characterized by emotional detachment, inconsistent discipline, or a lack of warmth, undermines social bonds and weakens commitment to conventional norms, thereby heightening susceptibility to deviant behavior (Hoeve et al., 2009; Amran and Basri, 2020; Jacobsen and Zaatut, 2022). In effect, dysfunctional parent–child relationships not only impair psychological well-being, social adjustment, and healthy personality formation but also foster relational contexts that normalize or reinforce aggression and antisocial behavior (Delgado et al., 2022). As familial influence wanes, adolescents often seek alternative sources of belonging and identity, increasing their affiliation with deviant peer networks, a shift that substantially amplifies delinquency risk (Allen et al., 2025). Both classical and contemporary scholarship consistently identifies weak or conflictual parent–child relationships as a strong predictor of subsequent juvenile offending (Gottfredson and Hirschi, 1990; Hoeve et al., 2009).

In summary, parent–child relationships constitute a vital socialization context and function as a key protective factor against juvenile delinquency. The strength of an adolescent's bond with parents is positively associated with the internalization of prosocial norms and resilience against negative peer and environmental influences (Delgado et al., 2022). Supportive and engaged parenting fosters prosocial development and reduces delinquency risk by reinforcing youths' attachment to conventional values and social rules (Hirschi, 2002; Schroeder et al., 2010). Thus, relationships characterized by emotional warmth, consistent behavioral monitoring, and positive reinforcement serve as effective buffers against deviant and criminal involvement. Grounded in this theoretical framework, the following hypothesis is proposed:

*H1*: Positive parent–child relationships are negatively associated with juvenile delinquency; that is, better parent–child relationships correspond to lower likelihood of juvenile delinquency.

### 2.2 The mediating role of self-control competence

Self-control is defined as the individual capacity to resist immediate impulses, manage temptations, and stay focused on long-term goals. In criminological theory, Gottfredson and Hirschi's (1990) general theory of crime posits that low self-control constitutes a primary etiological factor underlying deviant and criminal behavior, an argument supported by extensive research linking self-control deficits to a wide range of antisocial outcomes (Vazsonyi et al., 2017). This body of work also highlights the developmental foundations of self-control, emphasizing its origins in early socialization processes, particularly within the family context. Consistent evidence indicates that inadequate or ineffective parenting practices are robustly associated with impairments in self-regulation, which in turn foster lower levels of self-control (Janssen et al., 2016).

Self-control competence is fundamentally linked to behavioral regulation. Individuals with lower self-control are more susceptible to immediate temptations and peer influence, often prioritizing short-term rewards over long-term benefits, which elevates their risk of engaging in delinquent and criminal conduct (Gottfredson and Hirschi, 1990; Hay, 2001; Courey and Pare, 2016). The development of self-control is also strongly influenced by parent–child relationships. Secure attachment and effective parental supervision facilitate the capacity for delayed gratification and emotional regulation, whereas deficient parent–child interaction undermines self-control and promotes impulsive tendencies (Vazsonyi and Belliston, 2007; Cho et al., 2017).

As a result, self-control operates as a key psychological mechanism through which family dynamics influence delinquency. Empirical studies indicate that supportive and engaged parent–child relationships are associated with higher levels of self-control in adolescents, which in turn attenuates involvement in delinquent behavior (Fix et al., 2021; Guo, 2018). Moreover, the relationship between self-control and delinquency is moderated by gender. Females generally report higher average self-control, yet the protective effect of self-control against aggression and delinquency appears more pronounced among males (Hamama and Hamama-Raz, 2021).

In summary, self-control functions as a mediating pathway between parent–child relational dynamics and juvenile delinquency, with evidence suggesting that its role may vary across genders. Based on the foregoing review, the following hypotheses are proposed:

*H2*: Self-control competence mediates the relationship between the parent–child relationship and juvenile delinquency.

*H3*: The relationship between self-control competence and juvenile delinquency differs by gender, with stronger effects observed among males.

### 2.3 The mediating role of deviant peer association

Deviant peer association is widely acknowledged as a key proximate factor in the etiology of juvenile delinquency (Nisar et al., 2015; Zhu et al., 2016). During adolescence, peer influence is particularly salient; youth frequently conform to group norms to gain social acceptance, and when these norms deviate from conventional standards, they may be drawn into antisocial or criminal conduct (Barnes et al., 2006; Rokven et al., 2017). Moreover, adolescents tend to associate with peers who exhibit similar behavioral tendencies, a process of homophily that reinforces the formation and stability of delinquent peer groups (Ragan, 2020).

Parent–child relationships play a critical role in shaping these peer interactions. When attachment to parents is weak or strained, adolescents may experience emotional dissatisfaction and turn to peers for support, increasing their exposure to deviant peer networks (Worthen, 2011). Such exposure has been consistently associated with elevated risks of delinquency and related problem behaviors (Warr, 2005; Ray and Park, 2024). Conversely, higher levels of parental care, understanding, and effective supervision strengthen the parent–child bond, which can reduce adolescents' propensity to affiliate with deviant peers and buffer against the negative influences that contribute to delinquent behavior. Based on this theoretical and empirical foundation, we propose the following hypothesis:

*H4*: Deviant peer association mediates the relationship between the parent–child relationship and juvenile delinquency.

### 2.4 Self-control competence, deviant peer association and juvenile delinquency

Gottfredson and Hirschi (1990) posit that individuals with low self-control are more susceptible to engage in deviant behavior, especially when they affiliate with delinquent peers. Those with lower self-control are prone to irresponsible or undesirable conduct (Chen et al., 2022), and their impulsivity and self-centered tendencies often generate interpersonal conflicts with peers (Boman IV et al., 2019). Such relational difficulties can undermine stable friendships and increase the likelihood of peer rejection (Demuth and Brown, 2004; Chapple, 2005). Following rejection, adolescents may gravitate toward delinquent peer groups, which are often more tolerant of norm-violating behavior (Warr, 2002). This process can be summarized as a sequential pathway: low self-control → peer conflict/rejection → association with deviant peers → delinquent behavior.

**Figure 1:**
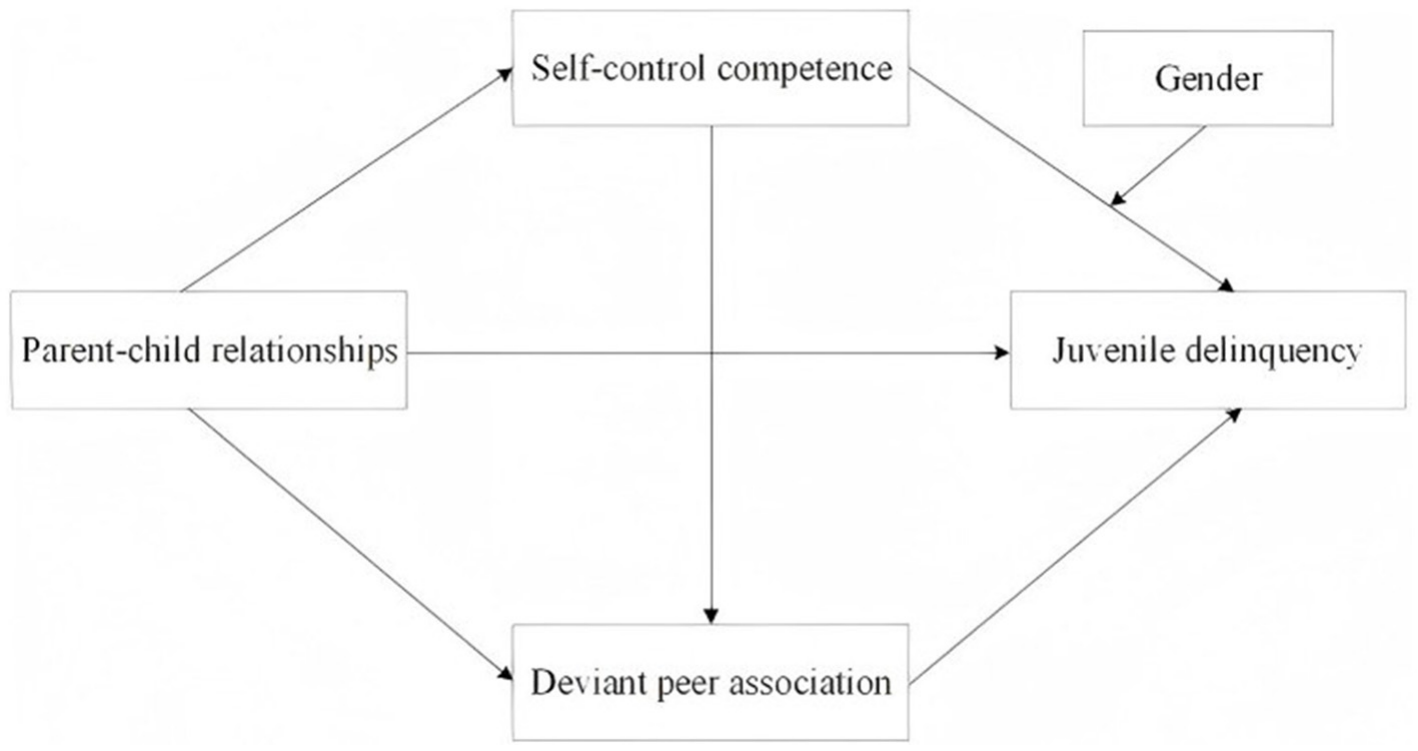
The serial relationship between self-control competence, deviant peer association, parent–child relationships, and juvenile delinquency.

The family, as the primary context of early socialization, plays a critical role in shaping adolescent conduct through parent–child relationships. These relationships influence both the development of self-control and the nature of peer interactions, thereby mitigating the risk of delinquency. Specifically, supportive and engaged parent–child relationships foster greater self-control competence and reduce the propensity to associate with delinquent peers. Conversely, weak parental attachment and tenuous social bonds increase reliance on deviant peer networks and heighten the likelihood of engaging in delinquent acts (Hirschi, 2002). Taking together, this corpus of research indicates a credible sequential mechanism connecting family relational dynamics, self-control, deviant peer association, and juvenile delinquency. Accordingly, we propose a serial mediation hypothesis, with the specific conceptual pathway illustrated in Figure 1.

*H5*: Parent–child relationships are associated with juvenile delinquency through the serial mediating effects of self-control competence and deviant peer association.

## 3 Materials and methods

### 3.1 Data sources

This study uses data from the 2019 National Ministry of Justice Recidivism Survey, a nationwide investigation that collected information through structured interviews, questionnaires, and standardized scales administered to incarcerated individuals across selected provinces and municipalities in China. To ensure national representativeness, a multi-stage stratified sampling design was employed. The country was divided into seven geographic-economic regions: North China, East China, Central China, South China, Northeast China, Northwest China, and Southwest China. From these regions, 13 provinces and municipalities were systematically selected. Within each selected region, the following facility types were randomly sampled: two adult male prisons (one minimum-security and one maximum-security inmates), one juvenile detention center, one women's prison, and two community correction centers. Within each sampled facility, systematic sampling was applied to select participants from official inmate rosters for interview administration.

As the most recent nationally representative micro-level survey of the incarcerated population in China, this dataset provides a comprehensive empirical foundation for addressing the research questions. The original survey encompassed both first-time and recidivist offenders. After data cleaning and variable selection, the final analytic sample consisted of 2,055 first-time offenders, with 736 reporting that their first offense occurred between the ages of 12 and 25.

### 3.2 Variable design

#### 3.2.1 Dependent variable

The dependent variable is a binary indicator of whether the respondent's first recorded offense occurred between the ages of 12 and 25. In this study, offenses committed within this age range were classified as juvenile delinquency, whereas those occurring outside this interval were categorized as non-juvenile delinquency. This classification was derived from the survey item: “How old were you when you committed your first crime?” The age span of 12–25 was selected based on criminological and developmental research, which identifies the period from early adolescence through emerging adulthood (roughly the early twenties) as a phase characterized by ongoing physical maturation and heightened social pressures (Johnson et al., 2011; Arnett, 2023). During this developmental window, elevated rates of offending are often attributed to an imbalance between emerging self-regulatory capacities and salient external influences (Burt, 2020; Steinberg et al., 2008). By approximately age 25, the prefrontal cortex, a brain region critical for impulse control, decision-making, and long-term planning, reaches full neurobiological maturity, contributing to greater psychological stability and a more consolidated sense of self (Burt, 2020; Casey, 2015; Johnson et al., 2011; Montroy et al., 2016).

#### 3.2.2 Independent variable

The independent variable in this study is parent–child relationships. Respondents' retrospective perceptions of their relationship with their parents prior to their first offense were assessed using the survey item: “Before your first crime, what was your relationship with your parents like?” Consistent with prior research (Zeng et al., 2024; Rudy et al., 2023), higher scores reflect more positive parent–child relationships. The survey offered three response options: poor, average, and good, which were coded as 1, 2, and 3, respectively, for analytical purposes.

#### 3.2.3 Mediator variables

Two mediating variables were examined in this study: self-control competence and deviant peer association. Self-control competence was conceptualized as the capacity to resist immediate impulses and sustain goal-directed behavior. It was measured using the Brief Self-Control Scale (BSCS), an abbreviated adaptation of the original Self-Control Scale (SCS). The BSCS comprises two subdimensions, self-discipline and impulse control, and includes a total of seven items (Morean et al., 2014). The standardized Chinese version of this scale has been validated in adolescent populations in China and has demonstrated strong reliability and validity for assessing self-control (Haktanir et al., 2024). Higher scores on the BSCS reflect greater self-control competence, and the variable was treated as continuous in the analyses. In the present sample, the scale showed satisfactory internal consistency, with a Cronbach's α of 0.77.

Deviant peer association was operationalized as whether the respondent had peers involved in illegal or criminal activities prior to their first offense. This was assessed with the single-item measure: “Before you committed a crime for the first time, did any of your peers engage in illegal or criminal behavior?” Respondents who reported having such peers were coded as 1 (deviant peer association present), and those who did not were coded as 0 (no deviant peer association). This variable was treated as binary in all subsequent analyses.

#### 3.2.4 Control variables

At the individual level, several demographic and socioeconomic characteristics were included as control variables: gender, household registration type (hukou) at the time of the first offense, school dropout experience, only-child status, left-behind status during childhood, personal educational attainment (categorized as primary school or below, junior high school, and senior high school or above), and household income stability at the time of the first offense. At the family level, key socioeconomic controls consisted of the father's educational level (classified as primary school or below, junior high school, senior high school, and college or above) and the father's occupational sector (categorized as public sector or private sector employment).

### 3.3 Methods

In this study, all statistical analyses were performed using Stata 18. Binary logistic regression was employed to examine the direct association between parent–child relationships and juvenile delinquency, as well as the mediating roles of self-control competence and deviant peer association. To test the hypothesized serial mediation pathway, in which self-control competence and deviant peer association sequentially mediate the relationship between parent–child relationships and delinquency, a structural equation modeling (SEM) framework was adopted. SEM allows for the simultaneous estimation of multiple direct and indirect pathways, providing a more integrated and nuanced assessment of the relationships among parent–child dynamics, self-control, peer influence, and delinquency. The significance of the indirect (mediation) effects was evaluated using a non-parametric bootstrap procedure with 1,000 resamples. Mediation was considered statistically significant if the bias-corrected 95% confidence intervals did not include zero. For moderating variables, simple slope analyses were conducted to probe interaction effects.

## 4 Results

### 4.1 Correlation analysis of variables

A Pearson correlation analysis was conducted for all study variables, with results summarized in Table 1. The control variables included gender, only-child status, household registration type (hukou), left-behind experience, personal educational attainment, household income stability, father's education level, and father's occupational sector. The key findings are as follows: (1) Both parent–child relationships (*r* = −0.247, *p* < 0.01) and self-control competence (*r* = −0.200, *p* < 0.01) were significantly negatively correlated with juvenile delinquency. In contrast, deviant peer association showed a significant positive correlation with delinquency (*r* = 0.202, *p* < 0.01). (2) Gender was significantly associated with delinquency (*r* = 0.236, *p* < 0.01), indicating that males reported higher levels of delinquent involvement. (3) Parent–child relationships were positively correlated with self-control competence (*r* = 0.183, *p* < 0.01) and negatively correlated with deviant peer association (*r* = −0.116, *p* < 0.01). (4) Self-control competence was negatively associated with deviant peer association (*r* = −0.164, *p* < 0.01). Overall, these correlational patterns provide preliminary support for the hypothesized relationships in this study.

**Table 1:** Correlation analysis of variables.

Variables	*M*	*SD*	1	2	3	4	5	6	7	8	9	10	11	12
Gender	0.729	0.444	1											
Only child status	0.776	0.417	−0.054^**^	1										
Type of household	0.631	0.483	0.181^***^	0.178^***^	1									
Left-behind status	0.112	0.315	0.088^***^	−0.024	0.150^***^	1								
Personal education attainment	1.143	0.749	−0.042^*^	−0.189^***^	−0.405^***^	−0.070^**^	1							
Household income stability	0.631	0.483	−0.073^**^	−0.005	−0.192^***^	−0.068^**^	0.212^***^	1						
Father's education level	0.608	0.824	−0.070^**^	−0.166^***^	−0.300^***^	−0.170^***^	0.358^***^	0.145^***^	1					
Father's occupation	0.116	0.321	−0.046^**^	−0.052^**^	−0.348^***^	−0.110^***^	0.268^***^	0.148^***^	0.355^***^	1				
Parent–child relationships	2.671	0.574	−0.067^**^	0.033	−0.130^***^	−0.256^***^	0.163^***^	0.196^***^	0.143^***^	0.100^***^	1			
Self-control competence	20.277	3.686	−0.005	0.051^**^	−0.138^***^	−0.113^***^	0.167^***^	0.184^***^	0.140^***^	0.127^***^	0.183^***^	1		
Deviant peer association	0.372	0.483	0.122^***^	−0.006	0.082^***^	0.113^***^	−0.089^***^	−0.151^***^	−0.058^**^	−0.084^***^	−0.116^***^	−0.164^***^	1	
Juvenile delinquency	0.358	0.480	0.236^***^	−0.104^***^	0.246^***^	0.260^***^	−0.072^**^	−0.279^***^	−0.090^***^	−0.198^***^	−0.247^***^	−0.200^***^	0.202^***^	1

*N* = 2,055, ^*^p < 0.1, ^**^p < 0.05, ^***^p < 0.01.

### 4.2 Baseline regression results

The baseline regression results, presented in Table 2, indicate a significant negative association between parent–child relationships and juvenile delinquency. In Model 1 (without control variables), both average (β = −1.059, *p* < 0.01) and good (β = −1.878, *p* < 0.01) parent–child relationships were significantly associated with a lower likelihood of delinquency relative to the reference category (poor relationship). After adjusting for demographic and socioeconomic covariates in Model 2, the negative association remained robust: average (β = −0.774, *p* < 0.01) and good (β = −1.304, *p* < 0.01) parent–child relationships continued to predict significantly lower odds of juvenile delinquency.

**Table 2:** Regression results of the association between parent–child relationships and juvenile delinquency.

Variables	Model 1	Model 2	Model 3	Model 4
	Juvenile delinquency	Juvenile delinquency	Juvenile delinquency	Juvenile delinquency
Male (female = 0)	–	1.039^***^ (0.142)	1.084^***^ (0.144)	0.997^***^ (0.143)
Only child (yes = 0)	–	−0.700^***^ (0.137)	−0.653^***^ (0.138)	−0.703^***^ (0.138)
Household registration (non-agricultural = 0)	–	0.758^***^ (0.134)	0.754^***^ (0.136)	0.784^**^ (0.136)
Left-behind status (no = 0)	–	1.289^***^ (0.184)	1.237^***^ (0.184)	1.226^***^ (0.187)
Personal education (primary or below = 0)	–	–	–	–
Junior high	–	−0.955^***^ (0.145)	−0.962^***^ (0.145)	−0.932^***^ (0.146)
Senior high or above	–	−0.479^***^ (0.176)	−0.536^***^ (0.176)	−0.513^***^ (0.177)
Stable income (unstable = 0)	–	−1.022^***^ (0.113)	−0.963^***^ (0.114)	−0.980^***^ (0.114)
Father's education (primary or below = 0)	–	–	–	–
Junior high	–	−0.316^**^ (0.131)	−0.305^**^ (0.132)	−0.282^**^ (0.132)
Senior high	–	−0.306^*^ (0.172)	−0.357^**^ (0.172)	−0.301^**^ (0.172)
College or above	–	0.524 (0.412)	0.636 (0.410)	0.570 (0.431)
Father's occupation (private sector = 0)	–	−1.305^***^ (0.236)	−1.304^***^ (0.237)	−1.309^***^ (0.242)
Parent–child relationships (poor = 1)	–	–	–	–
Average	−1.059^***^ (0.233)	−0.774^***^ (0.285)	−0.751^***^ (0.282)	−0.718^**^ (0.288)
Good	−1.878^***^ (0.221)	−1.304^***^ (0.271)	−1.220^***^ (0.268)	−1.240^***^ (0.274)
Self-control competence	–	–	−0.074^***^ (0.015)	–
Deviant peer association (no = 0)	–	–	–	0.524^***^ (0.112)
Intercept term	0.993^***^ (0.214)	1.285^***^ (0.385)	1.527^***^ (0.467)	1.219^***^ (0.393)
*N*	2,055	2,055	2,055	2,055
Pseudo *R*^2^	0.0457	0.219	0.228	0.228

Numbers in parentheses represent standard errors; ^*^p < 0.1, ^**^p < 0.05, ^***^p < 0.01.

To further examine the mechanisms underlying this association, we tested the distinct mediating roles of self-control competence and deviant peer association. When self-control competence was added as a mediator in Model 3, it showed a significant negative relationship with delinquency (β = −0.074, *p* < 0.01), while the protective effect of a good parent–child relationship remained significant. Similarly, in Model 4, deviant peer association was positively associated with delinquency (β = 0.524, *p* < 0 0.01), yet the negative coefficient for a good parent–child relationship persisted. These results suggest that both self-control and peer affiliation serve as relevant pathways in the relationship between parent–child relationships and delinquency, though they do not fully account for it.

### 4.3 Moderated serial mediation analysis

We employed SEM to examine the serial mediation pathway linking parent–child relationships quality to juvenile delinquency through self-control competence and deviant peer association. The path estimates are summarized in Table 3.

**Table 3:** Results for serial and moderated mediation pathways.

Variables	Model 5	Model 6	Model 7	Model 8
	Self-control competence	Deviant peer association	Juvenile delinquency	Juvenile delinquency
Male (female = 0)	0.384^**^ (0.180)	1.631^***^ (0.157)	1.042^***^ (0.145)	1.309^***^ (0.208)
Parent–child relationships (poor = 1)	–	–	–	–
Average	0.833^**^ (0.356)	−0.489^**^ (0.247)	−0.700^**^ (0.284)	−0.776^***^ (0.269)
Good	1.283^***^ (0.442)	−0.648^***^ (0.237)	−1.167^***^ (0.270)	−1.235^***^ (0.257)
Self-control competence	–	−0.059^***^ (0.015)	−0.067^***^ (0.016)	−0.052 (0.249)
Deviant peer association (no = 0)	–	–	0.474^***^ (0.112)	0.502^***^ (0.111)
Self-control competence × gender	–	–	–	−0.548^**^ (0.277)
Intercept term	16.944^***^ (0.617)	1.101^***^ (0.447)	1.174^**^ (0.479)	1.102^***^ (0.407)
Controls	Yes	Yes	Yes	Yes
*N*	2,055	2,055	2,055	2,055
Pseudo *R*^2^	0.0943	0.140	0.235	0.232

Numbers in parentheses represent standard errors; ^*^p < 0.1, ^**^p < 0.05, ^***^p < 0.01.

First, Models 5 and 6 tested the associations of parent–child relationships with the two proposed mediators. In Model 5, after controlling for covariates, respondents reporting good (β = 1.283, *p* < 0.01) or average (β = 0.833, *p* < 0.05) parent–child relationships demonstrated significantly higher self-control competence than those reporting poor relationships. In Model 6, relative to the poor-relationship group, respondents with average parent–child relationships showed a 38.7% lower likelihood of deviant peer association (β = −0.489, *p* < 0.05), while those with good relationships showed a 47.7% reduction (β = −0.648, *p* < 0.01). Self-control competence was also negatively associated with deviant peer association (β = −0.059, *p* < 0.01).

Second, Model 7 tested the full serial mediation model by incorporating both mediators simultaneously. After accounting for self-control competence and deviant peer association, average (β = −0.700, *p* < 0.05) and good (β = −1.167, *p* < 0.01) parent–child relationships remained negatively associated with delinquency. Notably, these coefficients were attenuated relative to those in Model 2, which excluded the mediators (average: β = −0.774, *p* < 0.01; good: β = −1.304, *p* < 0.01). Within Model 7, self-control competence continued to show a significant negative association with delinquency (β = −0.067, *p* < 0.01), while deviant peer association exhibited a significant positive association (β = 0.474, *p* < 0.01).

**Figure 2:**
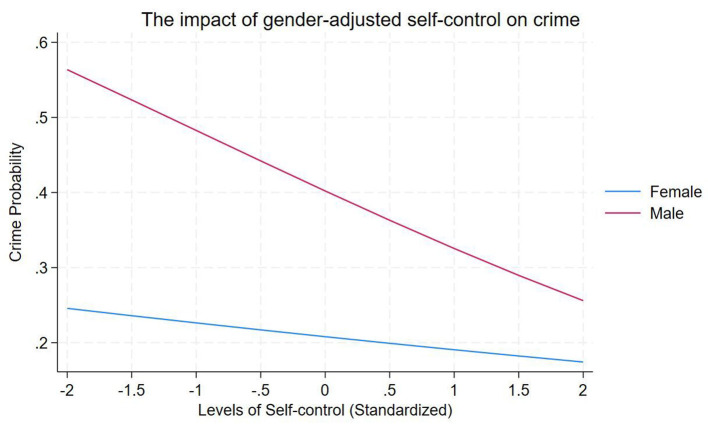
The moderating role of gender in the association between self-control competence and juvenile delinquency.

Finally, Model 8 introduced gender as a moderator of the link between self-control competence and juvenile delinquency. The interaction term between self-control and gender was significant (β = −0.548, *p* < 0.05). To facilitate interpretation, the marginal effects (simple slopes) of self-control were estimated separately for each gender. The results showed that self-control had a negative but non-significant effect among females (*dy/dx* = −0.019, *z* = −1.15, *p* = 0.250). In contrast, the effect was negative and statistically significant among males (*dy/dx* = −0.076, *z* = −6.37, *p* < 0.01). The difference in marginal effects between males and females was −0.057. This moderation effect is illustrated in Figure 2.

Building on the preceding analyses, this study further employed a bias-corrected non-parametric percentile bootstrap approach with 1,000 resamples to test the indirect pathways in the serial mediation model. Table 4 reports the 95% confidence intervals and effect proportions for all estimated paths. In the table, Path “a” denotes the total effect, Path “b” the direct effect, and Paths “c” through “e” represent specific indirect effects. All 95% confidence intervals excluded zero, confirming the statistical significance of each path.

**Table 4:** Mediated effect test.

Route	Effect value	Standard error	95% confidence interval		Proportion of total effect
			Boot LLCI	Boot ULCI	
(a) Total effect	−0.152^***^	0.019	−0.189	−0.115	–
(b) Parent–child relationships → juvenile delinquency	−0.112^***^	0.018	−0.147	−0.076	73.68%
(c) Parent–child relationships → self-control competence → juvenile delinquency	−0.005^**^	0.002	−0.010	−0.001	3.29%
(d) Parent–child relationships → deviant peer association → juvenile delinquency	−0.032^***^	0.008	−0.047	−0.017	21.06%
(e) Parent–child relationships → self-control competence → deviant peer association → juvenile delinquency	−0.003^***^	0.001	−0.005	−0.001	1.97%

^*^p < 0.1, ^**^p < 0.05, ^***^p < 0.01.

The decomposition of effects indicates that the total effect of parent–child relationships on juvenile delinquency was −0.152. The direct effect was −0.112, accounting for 73.68% of the total effect. The total indirect effect was −0.040, explaining 26.32% of the total effect. This indirect effect comprises three distinct pathways: the indirect effect via self-control competence alone, the indirect effect via deviant peer association alone, and the serial indirect effect passing sequentially through self-control competence and then deviant peer association. Specifically, the independent mediation through self-control competence accounted for 3.29% of the total effect, while the independent mediation through deviant peer association accounted for 21.06%. The serial mediation pathway explained an additional 1.97% of the total effect.

## 5 Discussion

This study systematically examines the relationship between parent–child relationships and juvenile delinquency, testing the mediating roles of self-control competence and deviant peer association, as well as the moderating role of gender in the link between self-control and delinquency. Using microdata from the 2019 National Recidivism Survey conducted by China's Ministry of Justice, the findings indicate that parent–child relationships are significantly associated with juvenile delinquency through both direct and indirect pathways. Specifically, we identified two independent mediating routes, through self-control competence and through deviant peer association, as well as one serial mediation pathway in which self-control and deviant peer association operate sequentially. Moreover, gender moderates the relationship between self-control and delinquency, with self-control exerting a stronger protective effect for males than for females.

These pathways align with core propositions from attachment theory, social control theory, self-control theory, and social learning theory. A secure parent–child bond appears to be associated with the development of self-regulatory capacities in adolescents. Conversely, when this bond is weak or compromised, adolescents' self-control tends to diminish, possibly contributing to their seeking affiliation with deviant peers. In this way, individual traits and social environmental risks interact, collectively increasing the likelihood of delinquent involvement. The following sections discuss these findings in greater detail.

### 5.1 Parent–child relationships and juvenile delinquency

Empirical evidence from this study demonstrates a significant negative association between positive parent–child relationships and juvenile delinquency. After adjusting for demographic and socioeconomic covariates, respondents reporting average or good parent–child relationships exhibited substantially lower odds of delinquency compared to those reporting poor relationships. This finding not only supports Hypothesis 1 but also aligns with prior research suggesting that a strong parent–child bond may function as a protective factor in adolescent development. Such bonds are associated with lower levels of internalizing issues like depression and loneliness and with a lower likelihood of engaging in deviant and criminal behavior (Vieira et al., 2016; Smetana and Rote, 2019).

Social control theory offers a useful framework for interpreting this association. It posits that the family constitutes the primary context of socialization, and that adolescents' emotional attachment to parents may be related to their internalization of societal norms (Hirschi, 2002). Positive parent–child relationships are associated with psychological security, a sense of belonging, and responsibility, while also encouraging open communication and reducing behavioral concealment (McAdams et al., 2017; Marciano et al., 2022). Conversely, weak or conflictual parent–child bonds are associated with weaker social ties and a greater likelihood of deviant conduct (Jacobsen and Zaatut, 2022; Hirschi, 2002).

However, several methodological considerations should be noted. First, parent–child relationships were assessed using a single item that categorized relationships as poor, average, or good. While this approach is efficient for large-scale surveys, it may not fully capture multidimensional aspects such as attachment, communication, supervision, and emotional support. Second, the retrospective nature of the assessment introduces the possibility of recall bias, as current experiences or post-offense reflections may influence perceptions of past relationships. Third, the sample consists exclusively of incarcerated individuals, which limits the generalizability to the broader juvenile population. Nevertheless, this design allows for an in-depth examination of the association between family dynamics and the onset of delinquency within a high-risk group.

To advance this line of inquiry, future research would benefit from incorporating community-based samples alongside multi-item, validated scales. Such approaches would offer a more nuanced understanding of the role of parent–child relationships across diverse youth populations and better capture the complexity of family dynamics in relation to delinquency.

### 5.2 Mediating roles of self-control competence and deviant peer association

Our study further examined the mediating roles of self-control competence and deviant peer association in the relationship between parent–child relationships and juvenile delinquency. Because the analytical sample consists exclusively of individuals with a criminal record, the outcome of interest is whether the first offense occurred between ages 12 and 25 rather than outside this age range. Accordingly, the mediation findings reported below should be interpreted with reference to whether the first offense occurred between ages 12 and 25 and understood as statistical associations rather than causal relationships. The results indicate that the direct effect of parent–child relationships accounts for the majority (73.68%) of the total effect on juvenile delinquency, indicating that it represents the predominant component of the observed association with adolescent offending. The indirect effect, while smaller in proportion (26.32%), is also statistically significant. Specifically, parent–child relationships are associated with delinquency not only directly, in relation to behavioral tendencies, but also indirectly through two sequential pathways: through their association with juveniles' self-control competence, and subsequently, with their association with deviant peers. This pattern is consistent with a set of associations in which personal traits and social relational contexts are jointly related to delinquent outcomes.

#### 5.2.1 Mediating role of self-control competence

The findings of this study support the mediating role of self-control competence in the association between parent–child relationships and juvenile delinquency, consistent with Hypothesis 2. Positive parent–child relationships are associated with higher self-control competence, which in turn is associated with lower juvenile delinquency. This aligns with prior research suggesting that family environments are associated with adolescent behavioral risk through their links with self-regulatory capacities (Fix et al., 2021). Self-control, a core component of self-regulation, develops prominently during childhood and early adolescence, a period in which regulatory abilities begin to consolidate (Montroy et al., 2016). This developmental process is closely related to parent–child interactions. Consistent emotional support and responsive parental guidance may be associated with stronger capacities in youth to delay gratification and regulate impulses (Jacobsen et al., 1997). Conversely, insecure attachment and inadequate supervision may be associated with lower levels of impulse control and a higher risk of delinquency (Vazsonyi and Belliston, 2007; Cho et al., 2017). Early emotional instability has also been linked to long-term difficulties in social and emotional functioning, which may in turn be associated with challenges in self-regulation and identity formation (Lanjekar et al., 2022).

Social control theory and the general theory of crime offer a theoretical basis for these patterns. Weak parent–child bonds are associated with weaker adolescents' attachment to family and broader society, lower internalization of social norms, and lower levels of self-control (Hirschi, 2002). Moreover, low self-control, conceptualized as a relatively stable trait, has been associated with a greater tendency to prioritize immediate rewards over long-term consequences and with a higher likelihood of criminal involvement (Gottfredson and Hirschi, 1990; Felson and Osgood, 2008). Together, these perspectives help explain how self-control may serve as one possible pathway in the association between family dynamics and delinquency.

It should be noted, however, that although the indirect effect through self-control is statistically significant, it accounted for only a modest portion of the total effect. The direct association between parent–child relationships and delinquency remains predominant, suggesting that self-control may represent only one of several factors involved in the association between family relationships and delinquent outcomes.

The study also revealed gender differences in the link between self-control competence and juvenile delinquency. Consistent with Hypothesis 3, the negative association between self-control and delinquency is stronger among males and is not statistically significant among females. This echoes previous research indicating that the negative association between self-control and delinquency tends to be more pronounced in males (de Kemp et al., 2008; Hamama and Hamama-Raz, 2021). One possible explanation may lie in gendered patterns of emotional and behavioral regulation: males often externalize distress through anger or acting out, whereas females are more likely to internalize problems as anxiety or depression (Kovess-Masfety et al., 2021). In this context, self-control may function primarily as a regulator of outward behavior, making it a particularly salient protective factor for boys. For girls, pathways to delinquency may involve more complex internal processes, such as emotional empathy or relational security, that were not fully examined in this study. Thus, this interpretation remains tentative and warrants further investigation. Overall, these findings suggest that gender may be an important consideration in prevention efforts, particularly in family-focused interventions and programs designed to enhance self-regulation.

#### 5.2.2 Mediating role of deviant peer association

The results also indicate that deviant peer association plays a mediating role in the association between parent–child relationships and juvenile delinquency, supporting Hypothesis 4. When deviant peer association was included in the model, the magnitude of the direct association between parent–child relationships and delinquency was attenuated, suggesting that peer affiliation may represent one pathway through which family dynamics are associated with delinquent outcomes. These finding bridges two established lines of inquiry: the association between parent–child relationships and peer selection (Warr, 2005; Worthen, 2011; Keijsers et al., 2011) and the association between peer relationships and problem behavior (Gifford-Smith et al., 2005; Reid et al., 2002). Prior research suggests that weaker parent–child bonds may be associated with greater reliance on peers for social identity and support, as well as with greater exposure to deviant peers and a higher likelihood of delinquency.

Both social learning theory and attachment theory offer useful frameworks for interpreting this pattern. Social learning theory posits that behavior is acquired through observation and reinforcement (Akers, 2017). From this perspective, adolescents surrounded by deviant peers are more likely to adopt and imitate deviant norms, especially when such behaviors are socially rewarded (Lu et al., 2020). Attachment theory, in turn, highlights the association between parental emotional support and secure attachment, which is linked to healthy emotional regulation and identity formation. Insecure attachment, which is often associated with poor parent–child relationships, has been linked to higher levels of depression and loneliness (Yildiz, 2016), which may amplify the salience of peer relationships as a source of validation and heighten susceptibility to peer influence (Warr, 2005; Ray and Park, 2024). However, the substitution or compensatory mechanisms implied by these theoretical perspectives were not directly examined in this study and therefore remain to be tested through formal interaction analyses.

#### 5.2.3 Serial mediation through self-control competence and deviant peer association

The results support a serial mediation pathway in which self-control competence and deviant peer association sequentially relate parent–child relationships to juvenile delinquency, consistent with Hypothesis 5. For respondents reporting average or good parent–child relationships, this sequential indirect pathway was associated with a lower likelihood of delinquency. Unlike prior studies that often examine isolated mediators, our findings highlight a more integrated sequence: parent–child relationships, a fundamental aspect of socialization, are associated with self-control competence, which in turn is associated with peer interaction patterns. Deviant peer association, as a more proximal social influence, appears to represent a closer correlate of delinquent involvement. These results suggest that family dynamics, individual traits, and peer contexts are interconnected in relation to delinquency rather than operating in isolation.

It should be noted, however, that the serial indirect pathway accounted for only 1.97% of the total effect, while the direct effect of parent–child relationships remained predominant (73.68%). Among the three indirect pathways, the route through deviant peer association contributed the largest share, followed by the pathway through self-control competence. This does not imply that self-control is unimportant; rather, it suggests distinct roles in the associations underlying delinquency. Self-control, as a relatively stable individual trait, may provide a baseline capacity for restraint. In contrast, deviant peer association operates as a more immediate external influence that can activate reward-seeking tendencies and thus exhibit a stronger proximal link to delinquent behavior (Burt, 2020; Steinberg et al., 2008). This pattern may be particularly salient in China's sociocultural context, where declining birth rates and the prevalence of single-child families have elevated the role of peers as sources of emotional support and belonging beyond the family. When parent–child bonds are weak, adolescents may become more oriented toward peer groups, potentially increasing exposure to deviant networks.

From a theoretical standpoint, adolescents with lower self-control may be more prone to impulsivity and immediate reward-seeking (Gottfredson and Hirschi, 1990), traits associated with greater difficulty in forming conventional peer relationships and a higher likelihood of affiliating with deviant peer groups (Demuth and Brown, 2004; Chapple, 2005). Within such groups, deviant behavior can be normalized and reinforced through social learning (Holt et al., 2012). Thus, deviant peer association may be understood not merely as an independent correlate of delinquency, but as a social context that may amplify pre-existing tendencies. While this process is consistent with the observed serial pathway, it remains inferential and warrants further direct examination.

Overall, these findings offer empirical support for an integrative perspective that bridges the general theory of crime (Gottfredson and Hirschi, 1990) and social learning theory (Akers, 2017). Rather than presenting competing explanations, self-control and peer influence appear to capture different stages of a broader pattern of associations. This contributes to ongoing debates about whether delinquency is more closely related primarily to stable individual traits or to person-context interactions. Importantly, parent–child relationships remain salient throughout the model: more positive relationships are associated with higher self-control, lower deviant peer affiliation, and, ultimately, a reduced likelihood of juvenile delinquency within this incarcerated sample.

## 6 Implications and limitations

Based on the findings of this study, several practical implications may be relevant to family-focused prevention of juvenile delinquency.

Firstly, given the observed association between parent–child relationships and juvenile delinquency risk, parents and guardians may benefit from prioritizing supportive family environments and nurturing strong emotional bonds with their children. Such bonds are associated with trust and connection, which may in turn be linked to fewer behavioral problems. Parents need not be parenting experts; rather, they should strive to serve as a primary source of support when children face stress, confusion, or temptation. This can be achieved through regular parent–child communication, improved parental listening skills, and the consistent expression of genuine care and affection.

Secondly, self-control competence, which in this study showed a significant mediating role in the association between parent–child relationships and delinquency, develops through stable, consistent, and predictable daily interactions. Parents may support self-regulation by honoring commitments to teach patience and delayed gratification, and by explaining the rationale behind rules rather than merely issuing commands. These practices may help children develop a stable behavioral framework that may support more reflective decision-making even in the absence of direct supervision.

Thirdly, the results highlight deviant peer association as the proximal factor most strongly associated with juvenile delinquency. Effective family intervention regarding peer networks may benefit from focusing not on direct monitoring or social restriction, but on indirectly supporting peer selection by strengthening children's self-control and ensuring they receive adequate emotional fulfillment within the family. When the home provides a reliable source of security and belonging, the psychological appeal of deviant peer groups may be reduced.

Finally, given that the negative association between self-control and juvenile delinquency was stronger among males, early and developmentally tailored interventions to enhance self-regulation are particularly valuable for boys. Such efforts should begin before conflicts intensify in early adolescence. Overall, gender differences warrant consideration in the design of targeted prevention strategies.

Although this study provides insights into the relationships among parent–child relationships, self-control competence, deviant peer association, and juvenile delinquency, several limitations warrant consideration. One limitation is that the cross-sectional design limits causal inference and precludes analysis of dynamic processes over time. Consequently, reverse causality, unobserved confounding, and the lack of temporal ordering cannot be ruled out. Additionally, key constructs, including parent–child relationships and deviant peer association, were measured using single-item retrospective indicators, which may not fully capture the multidimensional nature of family and peer dynamics. In addition, retrospective reports from incarcerated individuals may be subject to recall bias or reinterpretation shaped by current circumstances. A final limitation is that the exclusive reliance on an incarcerated sample restricts the generalizability of the findings to community-based adolescents and those not involved in the justice system.

Future research would benefit from longitudinal panel data to examine how these factors are associated with one another and vary across developmental stages. Employing validated multi-item scales would improve measurement precision, while mixed-methods approaches, combining surveys with in-depth interviews, could provide richer insight into the subjective and contextual dimensions of these associations.

## 7 Conclusion

Drawing on data from incarcerated individuals in China, this study employed a serial mediation model to provide empirical evidence on the pathways linking parent–child relationships, self-control competence, and deviant peer associations to juvenile delinquency. As a core component of family dynamics, parent–child relationships are found to be associated with juvenile delinquency both directly and indirectly. Specifically, compared to negative parent–child relationships, good and average relationships are associated with higher levels of self-control competence, more positive peer interactions, and a lower likelihood of juvenile delinquency. Notably, the negative association between self-control and juvenile delinquency appears stronger among males. These findings contribute to a more nuanced understanding of the factors associated with juvenile delinquency in the Chinese context and underscore the importance of parent–child relationships in adolescent socialization. Strong parent–child relationships are associated with higher self-control competence, which may in turn be linked to better emotional regulation and impulse management. With improved self-control, adolescents may be better able to make more reflective decisions when confronted with temptations, which may be associated with lower susceptibility to negative peer influences and early involvement in delinquency. This developmental process is especially salient during sensitive periods of adolescent growth. Consequently, interventions aimed at improving parent–child relationships, through practices such as open communication, consistent emotional support, active parental involvement, and stable guidance, may serve as a promising avenue for preventing juvenile delinquency. Such relationship-focused approaches not only strengthen familial bonds but also may support the internalization of self-regulatory skills associated with lower vulnerability to external risks.

## Data Availability

The datasets presented in this article are not readily available because they are not publicly accessible and were used under license. Requests to access the datasets should be directed to the China's Ministry of Justice.
